# Salmonid alphavirus replication in mosquito cells: towards a novel vaccine production system

**DOI:** 10.1111/1751-7915.12100

**Published:** 2014-01-14

**Authors:** Mia C Hikke, Marjan Verest, Just M Vlak, Gorben P Pijlman

**Affiliations:** Wageningen University, Laboratory of VirologyDroevendaalsesteeg 1, 6708 PB, Wageningen, The Netherlands

## Abstract

Salmonid alphavirus (SAV) causes pancreas disease and sleeping disease in Atlantic salmon (*Salmo* *salar*) and rainbow trout (*Oncorhynchus mykiss*) and confers a major burden to the aquaculture industry. A commercial inactivated whole virus vaccine propagated in a salmon cell line at low temperature provides effective protection against SAV infections. Alphaviruses (family *Togaviridae*) are generally transmitted between vertebrate hosts via blood-sucking arthropod vectors, typically mosquitoes. SAV is unique in this respect because it can be transmitted directly from fish to fish and has no known invertebrate vector. Here, we show for the first time that SAV is able to complete a full infectious cycle within arthropod cells derived from the Asian tiger mosquito *Aedes albopictus*. Progeny virus is produced in C6/36 and U4.4. cells in a temperature-dependent manner (at 15°C but not at 18°C), can be serially passaged and remains infectious to salmonid Chinook salmon embryo cells. This suggests that SAV is not a vertebrate-restricted alphavirus after all and may have the potential to replicate in invertebrates. The current study also shows the ability of SAV to be propagated in mosquito cells, thereby possibly providing an alternative SAV production system for vaccine applications.

## Introduction

Salmonid alphavirus (SAV) is a major burden in aquaculture of Atlantic salmon (*Salmo salar* L.) and rainbow trout (*Oncorhynchus mykiss* W.), and the causative agent of pancreas disease and sleeping disease. At present, at least six different SAV subtypes have been distinguished based on genotype and geographic distribution (Fringuelli *et al*., 2008; Graham *et al*., 2011; 2012). Infections may lead up to 48% mortality (McLoughlin and Graham, 2007) and deteriorated fillet quality (Lerfall *et al*., 2012). Salmonid alphavirus 3 (SAV3) is originally found in Norway, and infection of salmon results in loss of appetite, impaired swimming behaviour, and myopathy of heart and skeletal muscles (Hodneland *et al*., 2005). A commercial inactivated ‘whole virus’ vaccine propagated in a salmon cell line at low temperature (10–15°C) (Norvax® Compact PD, MSD Animal Health, Boxmeer, the Netherlands) provides effective protection against SAV infections. A prototype vaccine, based upon virus-like particles produced in *Spodoptera frugiperda Sf*9 insect cells by the baculovirus expression system, was recently developed. In this system, SAV3 envelope glycoproteins E1 and E2 were correctly folded when expressed at low temperatures and E1 retained fusogenic activity as observed by syncytia formation (Metz *et al*., 2011).

SAV3 is a unique member of the genus alphavirus (family of *Togaviridae*); the genome consists of a single, positive-stranded RNA of approximately 12 kb, which is 5′-capped and 3′-polyadenylated, allowing direct translation by the host cell machinery. Non-structural proteins (nsP1–4) are directly translated into one polyprotein that is processed into separate nsPs by the viral protease activity present in nsP2. Together the nsPs form a replication complex, allowing the synthesis of negative-stranded RNA and subsequent positive-stranded progeny viral RNA (Kuhn, 2007). In addition to the directly translated ‘non-structural’ polyprotein, a ‘structural’ polyprotein is translated from a subgenomic RNA, encoding the virion proteins capsid (C), envelope (E)3, E2, 6K and E1. Upon translation, C is autocatalytically cleaved off while proteolytic processing among E2, 6K and E1 occurs by host signalases in the endoplasmic reticulum. After translocation to the *trans*-Golgi system, host furin cleaves between E3 and E2, rendering the viral particle sensitive for low pH-induced activation (Kuhn, 2007).

Alphaviruses are generally characterised by the ability to transfer between hosts via blood-sucking arthropod vectors, typically mosquitoes. Well-known examples are Sindbis virus, Semliki Forest virus and chikungunya virus, the latter causing mainly fever, rash and arthralgia. Via their arthropod vector, they infect a wide range of vertebrate hosts including human and non-human primates (Jose *et al*., 2009). However, there are a few exceptions as well. Recently, Eilat virus replication was shown to be restricted to replication in mosquito cells and thus represents the first insect-only alphavirus (Nasar *et al*., 2012). In contrast, replication of SAV3 is limited to a small range of vertebrate fish cell lines, and it does not have a known invertebrate vector (Weston *et al*., 1999). Furthermore, no evidence of vertical transmission of SAV is available (Kongtorp *et al*., 2010). SAV was also shown to be effectively transmitted horizontally in cohabitation experiments, and this is assumed to be the main route of transmission in high-density aquaculture settings (Graham *et al*., 2011). Nonetheless, the haematophagous salmon louse *Lepeophtheirus salmonis* was found to contain the virus when caught feeding on the skin of an infected salmon, although no direct evidence of active viral replication within these arthropods was provided (Karlsen *et al*., 2006; Petterson *et al*., 2009). As outbreaks of SAV often coincide with a high sea lice burden in aquaculture tanks (Rodger and Mitchell, 2007), it is possible that salmon lice could serve as a transmission vector. If this is the case, the question is whether SAV is productively replicating within the sea lice and thus can be classified as an arthropod-borne (arbo)virus or not.

In this paper, it is shown for the first time that SAV is able to replicate within arthropod cells derived from the Asian tiger mosquito *Aedes albopictus*. This is the first indication that SAV is not a vertebrate-restricted alphavirus only and may have the potential to replicate in invertebrate hosts. The ability of SAV to replicate within mosquito cells provides a potential alternative system for SAV vaccine production.

## Results and discussion

**Figure 1:**
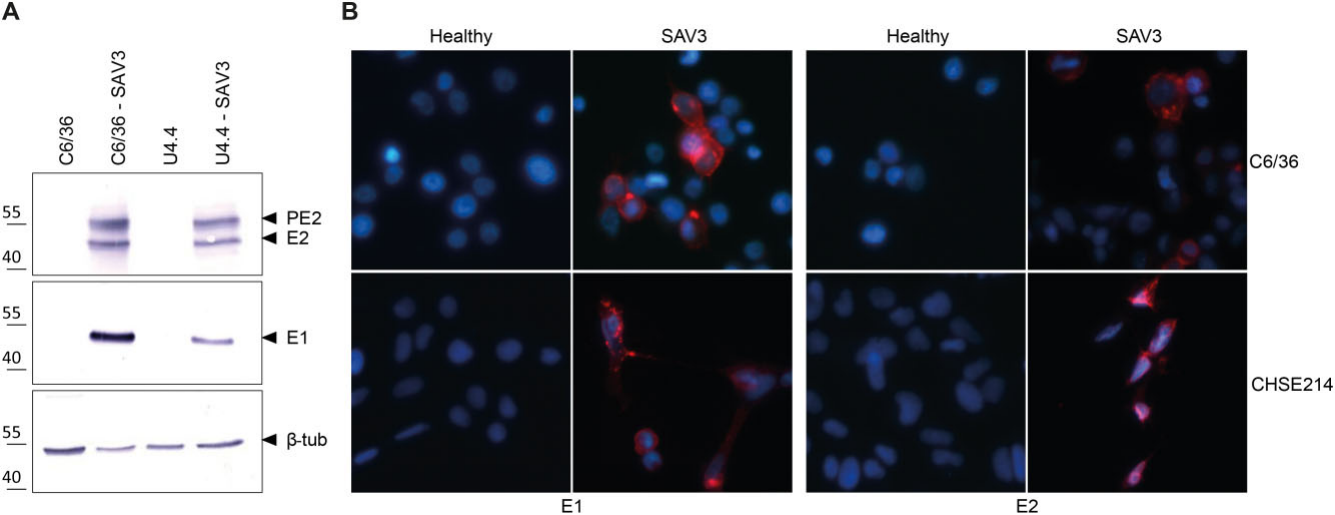
SAV3 infection of mosquito cell lines. A. Western blot analysis of proteins from invertebrate cell lines nine weeks after primary infection (15°C) with SAV3. Detection of SAV3 proteins presence was performed using mAb against SAV E1 (1:1000) and E2 (1:2000) glycoprotein. As loading control a polyclonal Ab against β-tubulin (1:2000, Abcam) was used. Protein sizes in kDa are indicated at the left. PE, precursor E2. B. Immunofluorescence detection of surface expressed SAV glycoproteins on C6/36 (upper panel) and CHSE-214 cells (lower panel). C6/36 and CHSE-214 cells were infected with SAV3 at a MOI 0.005 tissue culture infective dose 50% (TCID_50_) units cell^−1^ and incubated at 15°C for 10 days. E1 (left) and E2 (right) proteins are shown in red (Alexa-546). Hoechst 33258 nuclear staining (blue) was used to indicate cells.

At the moment, vaccination of young salmon parr/fingerlings occurs using adjuvanted, inactivated SAV virus from Chinook salmon embryo (CHSE-214) cells grown at low temperature. This low-temperature dependency of the production process makes upstream processing cumbersome and quite expensive. To examine alternative SAV propagation cell lines, a number of lepidopteran (*S. frugiperda* Sf9 and Sf21, *Trichoplusia ni* Hi5) and dipteran (*Culicoides* Kc, *Drosophila melanogaster* S2, *A. albopictus* U4.4 and C6/36) cell lines were infected with SAV3 and incubated at both 15°C and 18°C. These are the temperatures at which SAV replicates (15°C) or not (18°C) in salmonid CHSE-214 cells (McLoughlin and Graham, 2007; Graham *et al*., 2008). Replication of SAV3 in C6/36 and U4.4 mosquito cell lines at 15°C was observed when the proteins were analysed by sodium dodecyl sulphate polyacrylamide gel electrophoresis (SDS-PAGE) (10%) followed by Western blot detection of E1, precursor E2 and E2 protein using α-E1 (kindly provided by MSD Animal Health) and α-E2 [17H23 (Moriette *et al*., 2005)] monoclonal antibodies, respectively, in cell fractions at 4 weeks post-infection (wpi) [multiplicity of infection (MOI) ∼ 30] or 9 wpi (MOI 10) (Fig. 1A). At 15°C, the other cell lines did not show a positive signal for SAV3 proteins on Western blot (data not shown). In contrast, at 18°C, no virus replication was detected in any of the cell lines tested.

After infection of C6/36 cells at 15°C, progeny virus was harvested to infect fresh C6/36 cells. E2 was still detected in infected cell fractions after four serial, undiluted passages on the C6/36 mosquito cell line, suggesting that C6/36 cells support the complete SAV3 infectious cycle (entry, replication, assembly and secretion). Additionally, glycoprotein production and post-translational cellular transport in C6/36 cells was confirmed by surface immunofluorescence detection of the E1 (Fig. 1B, top left, 1:1000) and E2 (Fig. 1B, top right, 1:2000) glycoproteins. As a positive control, CHSE-214 cells were subjected to the same treatments as the C6/36 cells (Fig. 1B, bottom). Because the neutralizing 17H23 α-E2 mAb is expected to be a conformational antibody (Metz *et al*., 2011), detection of its epitope indicates correct folding of the E2 protein in C6/36 cells.

**Figure 2:**
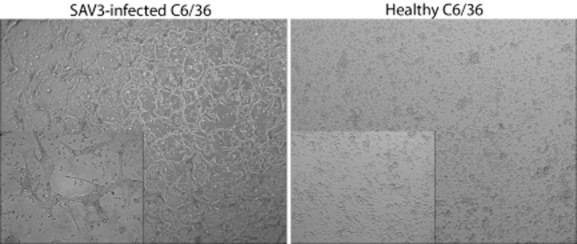
Syncytia formation of SAV3-infected C6/36 cells. SAV3-infected C6/36 cells (left) were incubated for 2 weeks at 15°C in cholesterol-enriched medium (0.1 mg ml^−1^). Infected and healthy cells were subjected to acidified (pH 5.5) medium for 2 min. Infected cells show syncytia formation 4 h post-acidification. Insets: magnification of the cell morphology.

A syncytia assay was performed to show that infection of C6/36 cells with SAV3 also results in the presence of a functional fusogenic E1 glycoprotein on the cell surface. Because alphavirus infection is cholesterol dependent and mosquitoes are cholesterol auxotrophs (Kielian and Helenius, 1984), incubation (15°C) was performed in cholesterol-supplemented medium (0.1 mg ml^−1^). Two weeks post-infection, the medium was removed and the cells were subjected to acidified medium (pH 5.5) for 2 min. Extensive syncytia formation was observed only in those samples infected with SAV3 (Fig. 2, left), confirming that the fusogenic activity of the E1 protein is retained when expressed during SAV3 replication in C6/36 cells. Thus, efficient functional glycoprotein production was observed in C6/36 cells. The virus could be serially passaged at least four times.

**Figure 3:**
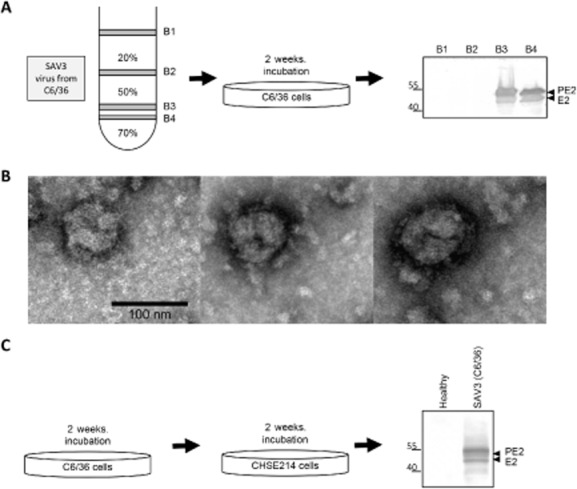
SAV3 grown in mosquito cells is still infectious to Salmonid cells. A. Medium of a SAV3 infection of C6/36 cells (2 weeks, 15°C) was fractionated by discontinuous sucrose gradient (20–70%) ultracentrifugation (2 h, 85 000 × g, 4°C). The four visible bands were used to infect fresh C6/36 cells (2 weeks, 15°C). Infected cell fractions were analysed using Western blot analysis using α-E2 mAb (1:2000). B. Transmission electron microscopy (JEOL JEM 1011, JEOL Ltd, Tokyo, Japan) of the sucrose cushion (20% w/v) concentrated SAV3-infected C6/36 cell culture medium fraction (2 h, 80 000 × g, 4°C). Samples were spotted on copper coated grids and stained with 2% uranyl acetate. C. SAV particles derived from C6/36 cells were used to infect Salmonid CHSE-214 cells. Infected cell fractions were analysed by Western blot analysis using α-E2 mAb (1:2000). Protein sizes in kDa are indicated at the left of the Western blot.

To further confirm the presence of *de novo* virion production, medium from SAV-infected C6/36 cell culture was fractionated via discontinuous sucrose gradient centrifugation (Fig. 3A). One band (B1) was visible on top of the 20% (w/v) sucrose layer, one band (B2) at the 20–50% (w/v) interphase and two bands (B3, B4) were observed around the 50–70% (w/v) sucrose interphase. The harvested bands were diluted 1:10 in Leibovitz (L15) medium, and each was used to infect fresh C6/36 cells. Analysis of this infection resulted again in detection of the E2 protein, indicating that the purified B3 and B4 sucrose fractions indeed contained infectious SAV3 particles (Fig. 3A).

An additional infection on C6/36 cells was performed to visualize C6/36-derived SAV particles from the medium by transmission electron microscopy. Analysis of sucrose purified samples showed particles of 60–85 nm in diameter (Fig. 3B), indistinguishable from SAV particles (Metz *et al*., 2011).

The *A. albopictus* cell line C6/36 is quite susceptible to viral infections and often used for *in vitro* cultivation of arboviruses (White, 1987; Sudhakaran *et al*., 2007; Arunrut *et al*., 2011). Most likely, this is due to a dysfunctional antiviral RNA interference (RNAi) system (Brackney *et al*., 2010). We have shown that C6/36 cells also support the full replication cycle of SAV3. The fact that also U4.4 cells, another *A. albopictus* cell line but with a functional RNAi system, are susceptible to SAV3 infection raises the question whether SAV replication in mosquito cells is under RNAi control (Sanchez-Vargas *et al*., 2004).

C6/36 cells are robust and fast-growing cells and easy to culture. Because they are lowly adherent by nature and do not need ethylenediaminetetraacetic acid or trypsin for detachment of monolayers from culture flasks, they are easily adaptable to growth in suspension (Morita and Igarashi, 1989). Additionally, no cooling equipment is needed for the proliferation of these cells (27–28°C) in the absence of virus infection, and therefore, this may be cheaper in upstream processing. Even though the production of infectious SAV3 particles is still restricted to a low temperature (< 15°C), upscaling of C6/36 cells at higher temperatures (27–28°C) prior to infection may decrease vaccine production times significantly. It would be interesting to measure the viral titres of SAV3 after a number of passages on C6/36 cells in comparison with virion production in CHSE-214 cells and to examine whether or not virus production levels are comparable.

Further development of mosquito cells as platform for antigen (vaccine) production should be conducted in follow-up studies. Upon optimization of this insect cell-based SAV production system, it will become clear whether or not mosquito cells can successfully outcompete salmonid cells in one or more of the following aspects: SAV antigen yield and quality, production speed and cost, and protective immunogenicity in fish.

Mosquito cells were incubated with SAV3 for a long period of time. Therefore, it is possible that upon replication, mutations may have arisen or been selected that allowed easier viral entry into the cell and/or budding from the cell. To examine if C6/36-derived SAV3 particles were still infectious to CHSE-214 cells, the virus inoculum was used to infect fresh CHSE-214 cells at 15°C. Two weeks post infection, cells were harvested and analysed on SDS-PAGE and WB, resulting in E2 detection in SAV3 infected cells (Fig. 3C). Therefore, we concluded that SAV3 produced in mosquito C6/36 cells can still readily infect Salmonid CHSE-214 cells. In addition, an important epitope on the E2 glycoprotein is still recognized by a neutralizing, conformational monoclonal antibody in an immunofluorescence assay and on Western blot. Although not tested, it is therefore likely that SAV produced in C6/36 or U4.4 cells is also infectious for salmon and trout.

The observation that SAV is able to replicate in mosquito cells is interesting from an evolutionary point of view as well. Phylogenetic tree analysis of alphaviruses places SAV in its own clade far diverged from all other alphaviruses (Powers *et al*., 2001). Our results are in line with previous research though that suggested that alphaviruses may have a marine (Forrester *et al*., 2012) or invertebrate (Ventoso, 2012) origin.

In conclusion, we showed in our experiments here that SAV3, while not currently being classified as an arbovirus, also grows in invertebrate, i.e. mosquito cells. Besides the biological importance of this observation, the use of invertebrate cells provides a putative alternative production platform for SAV vaccines.
